# Using autoencoders as a weight initialization method on deep neural networks for disease detection

**DOI:** 10.1186/s12911-020-01150-w

**Published:** 2020-08-20

**Authors:** Mafalda Falcão Ferreira, Rui Camacho, Luís F. Teixeira

**Affiliations:** 1 Faculty of Engineering, University of Porto, Rua Dr. Roberto Frias, s/n, Porto, 4200-465 Portugal; 2 INESC TEC - Institute for Systems and Computer Engineering, Technology and Science, Porto, Portugal

**Keywords:** Cancer, Classification, Deep learning, Autoencoders, Gene expression analysis

## Abstract

**Background:**

As of today, cancer is still one of the most prevalent and high-mortality diseases, summing more than 9 million deaths in 2018. This has motivated researchers to study the application of machine learning-based solutions for cancer detection to accelerate its diagnosis and help its prevention. Among several approaches, one is to automatically classify tumor samples through their gene expression analysis.

**Methods:**

In this work, we aim to distinguish five different types of cancer through RNA-Seq datasets: thyroid, skin, stomach, breast, and lung. To do so, we have adopted a previously described methodology, with which we compare the performance of 3 different autoencoders (AEs) used as a deep neural network weight initialization technique. Our experiments consist in assessing two different approaches when training the classification model — fixing the weights after pre-training the AEs, or allowing fine-tuning of the entire network — and two different strategies for embedding the AEs into the classification network, namely by only importing the encoding layers, or by inserting the complete AE. We then study how varying the number of layers in the first strategy, the AEs latent vector dimension, and the imputation technique in the data preprocessing step impacts the network’s overall classification performance. Finally, with the goal of assessing how well does this pipeline generalize, we apply the same methodology to two additional datasets that include features extracted from images of malaria thin blood smears, and breast masses cell nuclei. We also discard the possibility of overfitting by using held-out test sets in the images datasets.

**Results:**

The methodology attained good overall results for both RNA-Seq and image extracted data. We outperformed the established baseline for all the considered datasets, achieving an average F_1_ score of 99.03, 89.95, and 98.84 and an MCC of 0.99, 0.84, and 0.98, for the RNA-Seq (when detecting thyroid cancer), the Malaria, and the Wisconsin Breast Cancer data, respectively.

**Conclusions:**

We observed that the approach of fine-tuning the weights of the top layers imported from the AE reached higher results, for all the presented experiences, and all the considered datasets. We outperformed all the previous reported results when comparing to the established baselines.

## Background

Cancer is a label for a group of diseases that is characterized by abnormal and continuous cell growth, with the potential to spread through its surrounding tissues and other body parts [1]. During 2018, cancer was the second leading cause of death globally, accountable for 9.6 million deaths, where around 70% were in developing countries [2]. Throughout the years, and given the evolution of techniques, technology, and treatments in medicine, cancer survival rates have been improving [3]. However, there are still some types that have survival rates of under 20%, such as pancreatic, esophagus, and liver cancers. Its prevalence makes it more crucial to correctly and accurately classify such diseases. For tackling this need, many research groups have been trying to help on accelerating cancer diagnosis, by experimenting and studying the application of machine learning algorithms to this problem [4].

When automatically classifying tumor samples, one approach is to analyze the samples derived molecular information, which is its gene expression signatures. Gene expression is the phenotypic manifestation of a gene or genes by the processes of genetic transcription and translation [5]. By studying it, this gene map can help to better understand cancer’s molecular basis, which can have a direct influence on this disease’s life cycle: prognosis, diagnosis, and treatment. There are two main cancer genomics projects — The Cancer Genome Atlas (TCGA) [6] and The International Cancer Genome Consortium (ICGC) [7] — that aim to translate gene expression, systematizing thousands of samples across different types of cancers. With this elevated number of features, each representing a particular gene, one may find genome-wide gene expression assays datasets in these projects. However, this type of data presents some challenges, because of (1) a low number of samples, (2) an unbalanced class distribution, with few examples of healthy samples, and (3) a high potential of underlying noise and errors, due to eventual technical and biological covariates [8]. This difficulty in gathering data accurately is underlying for every dataset creation. The equipment used to collect the data has intrinsic errors associated (mechanical, of acquisition, and others), hence, the dataset will reflect these errors.

Several authors have chosen the previously mentioned approach of analyzing the gene expression of tumor samples. Many of the developed methodologies in this scope use straightforward supervised training, especially when using deep neural networks (DNNs), relying on their depth to produce the best results. Gao et al. [9] proposed *DeepCC*, a supervised deep cancer subtype classification framework based on deep learning of functional spectra quantifying activities of biological pathways, robust to missing data. The authors conducted two studies, each with a different cancer detection (colorectal and breast cancer data). The authors claimed that the described method achieved overall higher sensitivity, specificity, and accuracy compared with other classical machine learning methods widely used for this kind of task, namely random forests, support vector machine (SVM), gradient boosting machine, and multinomial logistic regression algorithms, with an accuracy higher than 90%.

Sun et al. [10] proposed *Genome Deep Learning* (GDL), a methodology aiming to study the relationship between genomic variations and traits based on DNNs. This study analyzed over six thousand samples of Whole Exon Sequencing (WES) mutations files from 12 different cancer types from TCGA, and nearly two thousand healthy WES samples from the one thousand genomes projects. The main goal of GDL was to distinguish cancerous from healthy samples. The authors built: 12 models to identify each type of cancer separately, a total-specific model able to detect healthy and cancerous samples, and a mixed model to distinguish between all 12 types of cancer-based on GDL. All the experiments were evaluated through: (a) three performance metrics — accuracy, sensitivity, and specificity — and (b) Receiver Operating Characteristic curves, with the respective Area Under the Curve (ROC-AUC). This methodology achieved a mean accuracy of 97.47% on the specific models, 70.08% on mixture models, and 94.70% on total specific models, for cancer identification.

In [11], Kim et al. compared the performances of: (1) a neural network, (2) a linear SVM, (3) a radial basis function-kernel SVM, (4) a k-nearest neighbors, and (5) a random forest when identifying 21 types of cancers and healthy tissues. The classifiers were trained with RNA-seq and scRNA-seq data from TCGA, where they selected up to the 300 most significant genes expressed for each of the cancer variations. To determine the optimal number of genes for each classifier’s binary classification task, the methods mentioned above were trained with 12 different sizes of gene expression datasets (from 5 to 300 genes). When learning with 300 genes, the neural network, the linear SVM, and the radial basis function-kernel SVM models achieved their best performance, with a with a Matthews Correlation Coefficient (MCC) of 0.92, 0.80, and 0.83, respectively. The k-nearest neighbors and random forest models achieved an MCC of 0.8 and 0.83, accordingly, when using 200 genes. Furthermore, the authors identified 10 classes with an accuracy of over 90%, and achieved a mean MCC of 0.88 and a mean accuracy of 0.88, with the neural network classifier.

However, many DNNs, besides the known open challenges regarding their training setting [12], have a higher tendency to overfit, which one can detect when applying the same architecture to unseen data (or to a held-out test). Thus, our motivation focuses on exploring unsupervised pre-training methods based on a lower-dimensional latent representation with the usage of an autoencoder (AE). This approach is grounded in the hypothesis that (a) there is unessential information in high dimensionality datasets, and (b) the acquisition and processing errors potentially present in the dataset are discarded, contributing to a lower probability of overfitting [13]. Furthermore, pre-training AEs and using the learned weights as priors of the supervised classification task not just improves the model initialization, but also often leads to better generalization and performance [13]. This may be one of the reasons why AEs are found to be the most predominant strategy when analyzing RNA-Seq data [14].

To support our motivation and choices, we present some works that include unsupervised training in their methodologies. In [15], the authors designed a solution by combining a Multilayer Perceptron and Stacked Denoising Autoencoder (MLP-SAE), aiming to predict how good genetic variants can be a factor in gene expression changes. This model is composed of 4 layers (input, two hidden layers from the AEs, and output, and trained it to minimize the chosen loss function, the Mean Squared Error (MSE). The authors started by training the AEs with a stochastic gradient descent algorithm to later use them on the multilayer perceptron training phase as weight initialization; cross-validation was used to select the best model. The performance of the chosen model was compared with the Lasso and Random Forest methods and evaluated on predicting gene expression values for a different dataset. The authors concluded that their approach (1) outperformed both the Lasso and Random Forest algorithms (with an MSE of 0.2890 *versus* 0.2912 and 0.2967, respectively), and (2) was able to capture the change in gene expression quantification.

The authors in [16] described a study of four different methods of unsupervised feature learning — Principal Component Analysis (PCA), Kernel Principal Component Analysis (KPCA), Denoising AE (DAE), and Stacked Denoising AE — combined with distinct sampling methods when tackling a classification task. The authors focused on assessing how influential the input nodes are on the reconstructed data of the AE’s output, when feeding these combinations to a *shallow* artificial network trained to distinguish papillary thyroid carcinoma from healthy samples. The authors highlighted two different results, in their 5-fold cross validation experiment: the combination of a SMOTE [17] with Tomek links and a KPCA, was the one with the best overall performance, with a mean F_1_ score of 98.12, while the usage of a DAE achieved a mean F_1_ score of 94.83.

In [18] presented a stacked sparse autoencoder (SSAE) semi-supervised deep learning pipeline, applied to cancer detection using RNA-Seq data. By employing layer-wise pre-training and a sparsity penalty, this approach helps to capture more significant information from the known high dimensionality of RNA-Seq datasets, using the filtered information to the sequent classification task. The SSAE model was tested on three different TCGA RNA-Seq datasets — corresponding to lung, stomach, and breast cancers) — with healthy and cancerous samples, and compared it to four others classification methods: an SVM, a Random Forest, a neural network (supervised learning only), and a vanilla AE. The authors performed 5-fold cross validation and evaluated the model’s performance through four metrics: accuracy, precision, recall, and F_1_ score. The results show that the semi-supervised deep learning approach achieved superior performance over the other considered methods, with an average F_1_ score of 98.97% across the three used datasets.

The authors in [19] developed a methodology for detecting papillary thyroid carcinoma. They analyzed how the usage of AEs as a weight initialization method affected the performance of a DNN. Six types of AEs were considered: Basic AE, Denoising AE, Sparse AE, Denoising Sparse AE, Deep AE, and Deep Sparse Denoising AE. Before being integrated into the classifier architecture, all AEs were trained to minimize the reconstruction error. Subsequently, they were used to initialize the weights of the first layers of classification neural network (meaning that the AE layers become the top layers of the whole classification architecture), using two different strategies when importing the weights: (1) just the encoding layers, and (2) all the pre-trained AE. Moreover, in the training phase, the authors studied two different approaches when building the classifier: (a) fixing the weights of the AE and (b) allowing subsequent fine-tuning of all the network’s weights. The authors used stratified 5-fold cross-validation and evaluated the model through 6 distinct metrics: Loss, Accuracy, Precision, Recall, and F_1_ score. The authors reported that the overall best result was achieved through a combination of Denoising AE, followed by its complete import into the classification network, and by allowing subsequent fine-tuning through supervised training, yielding an F_1_ score of 99.61.

In [20], the authors present a transfer learning methodology, in which the main goal is to explore whether leveraging the information extracted from a large RNA-Seq data repository, with multiple cancer types, leads to extract important latent features that can help complex and specific prediction tasks, such as identifying breast cancer neoplasia. The authors used the TCGA PanCancer dataset, which is composed of approximately 11,000 RNA-Seq gene expression examples of 33 distinct tumor types. This data was split into two sets: breast cancer and non-breast cancer data. The non-breast data is firstly used to train the three selected architectures for this study: a sparse AE, a deep sparse AE, and a deep sparse denoising AE models. Then, the breast data is used to fine-tune the resulting AEs. After pre-training these models, the authors aim to predict the breast tumor intrinsic-subtypes, which is given by the PAM50 subtype information included in the clinical data included in the PanCancer data. The extracted features from the AE-based architectures are then fed as input to three different machine learning classifiers, namely Logistic Regression, Support Vector Machine, and a shallow Neural Network. To assess the deep AEs performance as feature extraction methods, the authors compared them to other classical feature extraction methods, combining them with the classification algorithms previously mentioned: ANOVA, Mutual Information, Chi-Squared, and PCA. A 10-fold cross validation was performed, and all the combinations were compared through the accuracy metric. The results showed the deep sparse denoising AE performs best when using the AE extracted features, where the combination with a shallow neural network leads to the best overall of 90.26% (±2.85).

In [21], Ferreira et al. used the same methodology described in [19] to discriminate different types of cancer, instead of distinguishing cancerous samples from healthy ones. In this case, they aimed to identify thyroid, skin, and stomach cancer correctly. Given that a Denoising AE was the AE that lead to the best results in previous studies, the authors chose to single it out, instead of the original 6. The rest of the experiments remained the same: 2 strategies for importing the pre-trained AE into the top layers of the classifier, two approaches when training the classifier to detect different types of cancer, same evaluation of the obtained results. Although in a different domain, the best outcome was reached with a combination of the same strategy and the same approach in the previous work [19], with an F_1_ score of 98.04, when identifying thyroid cancer.

## Methods

We extend the previously described work in [21] by assembling three different types of experiments, divided into two main parts, where we use three different AEs and five types of cancer samples. In the first one, we analyze the performance of a deep neural network (DNN), using the same pipeline to identify different types of cancer. In the second part, we choose one of the used AEs to assess how: (1) the variance of its latent vector dimension impacts the essential information capture and therefore possibly influencing the classifier’s performance, and (2) different data imputation strategies can influence the overall performance in the classification task. Moreover, we study if the network architecture is correlated with its overall performance, and how the model reacts when training with a different data type dataset. We built this pipeline in Python, using: the Numpy [22] and Pandas [23] packages for the data preprocessing step; the Keras deep learning library [24] running on top of TensorFlow and the Scikit-Learn [25] package to train and evaluate the models; and the Matplotlib [26] library for visualization. Additionally, we used an NVIDIA GeForce RTX 2080 Ti GPU, on a Ubuntu 18.04 operating system.

This section is organized as follows: “The data” subsection describes the used data and its inherent preprocessing. “Autoencoders” subsection overviews the AEs considered to this study. “Methodology” subsection outlines the pipeline, for each of the referred experiments. “Evaluation” subsection details how we evaluate the results to provide statistical evidence. Finally, “Baseline” subsection presents the established baseline results for all the used datasets.

### The data

In our experiments, we use two different types of data, which are described in the subsections that follow.

#### RNA-Seq data

We used five different RNA-Seq datasets, from The Cancer Genomes Atlas (TCGA) [6], each representing a type of cancer: thyroid, skin, stomach, breast, and lung. One can find a sample of the described data in Table 1. The datasets were downloaded from the cBioPortal [27], which gathers cancer-related data from different projects, including TCGA. To train DNNs, we need as many data as we can get. Ergo, our first criterion was to choose cancer types that had the highest number of examples. Additionally, we decided to gice priority to cancer types with high mortality and high incidence rates. We use the same thyroid, skin, and stomach datasets presented in [21], alongside the lung and breast datasets. The data filtering process in the cBioPortal comprised searching with the keywords *PanCancer*, sorting the obtained results from highest to lowest RNA-Seq examples, and finally selecting the thyroid, skin, stomach, breast, and lung datasets.

**Table 1:** Five instances of the thyroid RNA-Seq dataset we have used

	UBE2Q2P2	HMGB1P1	LOC155060	...	ZZZ3	TPTEP1	AKR1C6P
0	-1.6687	NA	NA	...	-0.9478	-1.3739	NA
1	-1.1437	NA	NA	...	-0.4673	-0.0166	NA
2	-0.9194	NA	NA	...	2.1918	-1.5856	NA
3	1.1382	NA	NA	...	1.5512	-1.5897	NA
4	-0.3333	NA	NA	...	0.4926	-1.3379	NA

The first line (the header) contains the genes names, and the column values represent its expression, sample-wise (except for the first column, which is the sample ID). *NA* stands for *missing value*, for a particular gene and sample

All five datasets are composed of approximately 20 thousand features. Each column feature in these datasets represents a specific gene, and the cell values for each column are the expression of that gene in a particular sample. All the RNA-Seq data were normalized according to the distribution based on all samples. The expression distribution of a gene is estimated by calculating the mean and variance of all samples with expression values, and discarding zero’s and non-numeric values such as *NA*, *Null* or *NaN*, which are substituted by *NA* [28]. With the five datasets, we gathered 509 examples of thyroid cancer, 472 of skin cancer, 415 of stomach cancer, 1,083 of breast cancer, and 511 of lung cancer. We would like to emphasize that this dataset is only a toy dataset since the data does not fairly reflect the immense difficulty associated with identifying cancer in a real scenario.

The preprocessing pipeline was executed for each RNA-Seq dataset separately. Firstly, we removed the columns that had only one value throughout all samples. When a value is constant for all the examples, there is no *entropic value*; with no value variation, one cannot infer any information. In total, 2,056, 2,072, 1,993, 457, and 591 columns were removed on the thyroid, skin, stomach, breast, and lung datasets, respectively. By default, we attributed the remaining missing values (represented by *NA* in the dataset, as observable in Table 1) with the mean value of the column where the missing value is [29]. Further normalization was not applied in the data. Finally, we added the *Label* column, to link the instances to their type of cancer, when training the classifier.

Since we aim to distinguish several cancer variations, we test all cancers against each other, assigning the positive value one to the class of interest, and zero to the remaining ones. When detecting thyroid cancer, all thyroid examples are labeled as one and the skin, stomach, breast, and lung instances as zero, and henceforward.

After processing all the datasets, it is improbable that the preprocessing phase removed the same columns in all of them. To guarantee the same features describe all the samples, we intersect all the datasets and use the result as our final dataset. Also, given that the breast cancer datasets had almost the double of instances, we apply downsampling and randomly select 500 breast cancer examples, to keep the final dataset as evenly distributed for all the cancers as possible. In the end, the resulting dataset has approximately 3,000 instances and more than 17 thousand genes.

#### Data of features extracted from images

We use two datasets of two different diseases, composed of features extracted from images: malaria and breast cancer. Since we aim to evaluate how well this methodology generalizes, by using distinct types of data, we are now able to gather evidence supporting this premise.

The malaria dataset was created by the Fraunhofer AICOS institution, through the MalariaScope project [30]. Their main goal is to develop low-cost solutions that can provide fast, reliable, and accurate results on detecting such disease, particularly in developing countries. In [31], the authors thoroughly describe the feature extraction process, from thin blood smear images exclusively acquired with smartphones. The resulting dataset is composed of 26,839 samples and 1,058 features. These features were normalized between [−1,1] via scaling and grouped into three main groups: geometry, color, and texture. From all the examples, approximately 8% contain malaria parasites. Due to the high unbalance between Malaria and Non-Malaria labels, we performed downsampling on the Non-Malaria class, where we randomly selected 60% examples. We decided to choose 60% instead of 50% due to a wide variety of non-parasite artifacts. Once the samples were selected, and similarly to the preprocessing step of the RNA-Seq data, we verify if there are features with constant values and remove them if that is the case. Our working malaria dataset has 5,906 instances (60% negative and 40% positive) and 1,052 feature columns.

The Wisconsin Breast Cancer dataset [32] from the UCI Machine Learning Repository is composed of 569 examples and 30 features. These features are computed from a fine needle aspirate digitized image of a breast mass and describe the cell nuclei characteristics present in those images, such as texture, area, concavity, and symmetry. From the 569 examples, approximately 60% are benign samples, and 40% are malign ones. No under or oversampling techniques were applied, since we do not find it to be needed. As performed in the malaria data, we checked if there were columns with constant values, for which there were not. The data was used as is, with the proportions and characteristics described above.

### Autoencoders

An autoencoder (AE) [33] is an unsupervised feature learning neural network, that aims to copy its input based on a lower dimensional representation. This type of architecture is able to extract features by reducing the dimension of its hidden layer [33], which helps the AE to focus on capturing the essential features that best represent the data.

Let the encoding and decoding functions of the AE be *f* and *g*, parameterized on *θ*_*e*_ and *θ*_*d*_ respectively, where *θ*=*θ*_*e*_∪*θ*_*d*_, *L* being the loss function, and *J* the cost function to be minimized. When learning, the AE aims to find value *θ* that:

1 $$\begin{array}{@{}rcl@{}}  \underset{\theta}{\text{argmin}}~J(\theta;X) = L(X, g_{\theta_{d}}(f_{\theta_{e}}(X)) \end{array} $$

penalizing the reconstruction of the input, given by $\hat {X}=g_{\theta _{d}}(f_{\theta _{e}}(X))$; the more distinct $\hat {X}$ is, the bigger the applied penalty. When training an AE, we use Mean Squared Error (MSE) as the loss function, and the Rectified Linear Units activation function (ReLU) [34] for all its layers. Currently, using ReLU as activation is the default recommendation, when training neural networks [35]. Similarly, using MSE as the loss function is a fairly common practice present in the literature, when training AEs [15, 35–37].

We use the AEs as a weight initialization technique [38] since evidence supports that using *“unsupervised pre-training guides the learning towards basins of attraction of minima that support better generalization from the training dataset”* [13]. Thus, we pre-trained them before importing the encoding part or all their layers to the classification neural network.

#### Basic autoencoder (AE)

The simplest AE has only one hidden layer. This type of AE learns through the optimization cost function presented in Eq. 1. With the combination of linear activations (ReLU) and the MSE loss function, these AEs behave similarly to the Principle Component Analysis (PCA) method — when trained with an MSE, an AE learns the principal subspace of the training data, consequentially [35].

#### Denoising autoencoder (DAE)

A Denoising AE (DAE) [39] aims not just to reproduce the input, but also to keep its information intact to undo the effect of an intentional corruption process applied to the original data. Its cost function can be described by:

2 $$\begin{array}{@{}rcl@{}}  \underset{\theta}{\text{argmin}}~J(\theta;X) = L(X, g_{\theta_{d}}(f_{\theta_{e}}(\tilde{X})) \end{array} $$

where $\tilde {X}$ is a copy of the input *X*, intentionally corrupted by a sort of *noise* [35]. To simulate a form of Bernoulli Noise [40], we apply a *Dropout* layer, immediately after the input layer, where 10 of the connections are randomly cut.

#### Sparse autoencoder

Similarly to a DAE, a Sparse AE (SAE) learning process also has two main goals: (1) minimizing the reconstruction error when aiming to copy the input data, and (2) applying a *sparsity penatly* (represented by *Ω* to the parameters involved in the encoding part:

3 $$\begin{array}{@{}rcl@{}}  \underset{\theta}{\text{argmin}}~J(\theta,X) = L(X, g_{\theta_{d}}(f_{\theta_{e}}(X + \lambda\cdot\Omega(\theta_{e}))) \end{array} $$

Although it also tries to reproduce *X*, an SAE can address unique statistical features of the dataset it has been trained on [35, 41]. To deliver that sparsity element, we use an L1 penalty, with a *λ* of 10^−5^.

### Methodology

We have adopted the methodology described in [19], which was also used in [21]. Our experiments consist of an analysis of the performance of a DNN, trained to classify different cancer types, studying how three different factors may impact the network performance:

1. The top layers, where we use three different AEs as weight initialization;
2. The dimension of the latent vector of the AEs, that means the encoding layer size;
3. The imputation technique, to replace missing data when preprocessing the datasets.

**Fig. 1:**
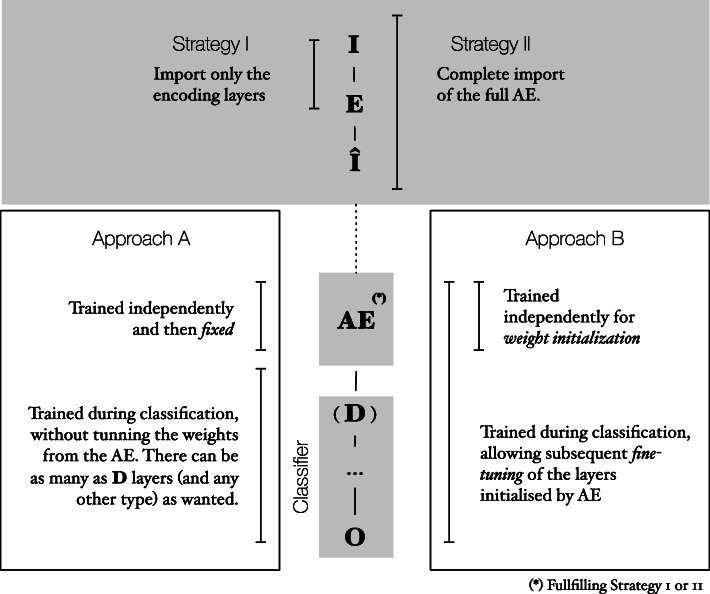
Overall pipeline of our experiments. This figure illustrates the chosen metodology for our work. Firstly, we pre-train the autoencoders (AEs), before embedding them to the top layers of the classification network, fullfilling either Strategy 1 (import only the encoding layers from the AE) or Strategy 2 (import the complete AE). Each of the full assembled architectures is then trained to detect one of the 5 cancer types, in the input data. The training process can follow two different approaches, regarding the imported weights of the AEs: (A) fixing them or (B) allowing subsequent fine-tune. *I* represents the input layer, *E* the encoding layer, $\hat {I}$ the output layer of the AE; at the classification region of the network, *D* represents the fully connected layer, and *O* the output of the classifer

For all these, we follow the same pipeline (see Fig. 1). For each experience, we start by pre-training a different AE to minimize the reconstruction error, before importing them into the top of the classification architecture. When doing so, we choose one of the two strategies considered for this study: (1) add just the encoding layers, or (2) add all the pre-trained AE. After the embedding of the AE to the top layers, we consider two different approaches in the training process: (A) fixing the imported weights of the AE layers, and (B) by allowing them to be fine-tuned, during the model training for the classification task.

With the complete architectures (AE as the top part of the classification network) assembled, we train each one to distinguish:

- The RNA-Seq input data as one of 5 cancers, namely thyroid, skin, stomach, breast, and lung;
- The malaria input data as Malaria or Non-Malaria;
- The breast masses input data as Malign or Benign.

Besides the top layers imported from the AE, the classification part of the full architecture is composed of a Batch Normalization layer [42], followed by two Fully Connected layers with a ReLU [34] activation. Since we aim to detect one type of cancer at the time, the last layer — the predictive one — is a single neuron layer with a Sigmoid non-linearity [43]. This activation considers that if the probability of the classification is lower than 0.5, the sample is classified as negative (that is not having the disease); otherwise, the sample is classified as positive.

To assess the following experiments, we decided to only use the AE that achieved the best results in the first experiments. For points (2) and (3), we try three different dimensions: 64, 32, and 16. For the data imputation study, we use three strategies: replacing the data with (a) the mean column value (used as default), a constant value (in this case, zero), and (b) with the most frequent value.

Furthermore, we want to study if when using Strategy 2 (importing the complete AE into the classification network) the model yields better results just because it has one more layer and, therefore, more parameters to train. To observe if the classifier is better only by being deeper, we pre-trained the AE and, at the embedding step for Strategy 1, we add a *decoder* layer, with all its weights randomized, guaranteeing that there are no discrepancies concerning the network’s topological complexity, for both strategies.

Finally, we want to assess how the pipeline behaves when dealing with different data types, besides RNA-seq entries. Hence, we apply the same methodology to the image extracted features datasets described in “The data” section, to assess if the model can adapt and generalize well to these data characteristics.

### Evaluation

We use stratified 10-fold cross-validation, to ensure and provide statistical evidence. The AEs are trained during 300 epochs, and the classifier during 500 with a batch size of 100. The classification model is trained with the binary cross-entropy loss function [35] and with an Adam optimizer [44]. Furthermore, we assess the overall performance of the model in the training and validation sets, by analyzing five more metrics: Accuracy, Matthews Correlation Coefficient (MCC) [45], Precision, Recall, and F_1_ score, and provide the Receiving Operator Curve, with the respective Area Under the Curve (ROC-AUC), and the Precision-Recall Curve.

Furthermore, to study how the model generalizes to unseen data during the training phase, we evaluate the performance of the best architecture combination on a held-out test set, for the Malaria and the Wisconsin Breast Cancer datasets. For both, and separately, we use a ratio of one third to create two new splits. Therefore:

- The Malaria train and test sets are composed of 3957 and 1949 examples, respectively;
- The Wisconsin Breast Cancer train and test sets are composed of 381 and 188 examples, respectively.

We performed a stratified split, meaning that we preserve the distribution of the label in both the train and test sets. With the training set, we followed the same stratified cross-validation strategy described above. The performance on the held-out set was assessed through the same metrics as well.

### Baseline

To support our claim that using AEs as weight initialization improves a DNN performance, we defined three different baselines, for each of the used datasets.

For the RNA-Seq data, we established as baseline the results from the classification part of our methodology, without the top layers of the AEs. The baseline model was trained under the circumstances described in the previous section. The results of such experiment can be found in Table 2, where the best overall performance was achieved when classifying skin cancer, with a mean F_1_ score of 51.15%.

**Table 2:** Baseline results for cancer detection, using a Fully Connected Neural Network (the classification architecture, without the AE as top layers)

	Accuracy (%)	MCC	Precision (%)	Recall (%)	F_1_ score
Thyroid	80.00 ±10.92	0.23 ±0.13	55.42 ±32.18	26.07 ±27.31	26.04 ±16.31
Skin	84.67 ±4.95	0.46 ±0.12	62.08 ±20.86	54.15 ±20.82	51.15 ±14.76
Stomach	81.33 ±11.47	0.27 ±0.18	56.66 ±32.34	33.85 ±27.06	30.94 ±17.66
Breast	85.60 ±2.07	0.34 ±0.16	80.95 ±21.33	25.60 ±17.51	33.99 ±19.01
Lung	77.13 ±12.59	0.21 ±0.18	39.90 ±37.04	33.73 ±35.76	25.25 ±22.47

All the presented results are the 10-fold cross-validation mean values, at the validation set, by selecting the best performing model according to its *F*_1_ *score*

We further added another baseline for the RNA-Seq datasets, where we use a simple AE with random and fixed weights, with the intent of discarding the possibility of our pipeline yielding better only because its classification architecture is slightly deeper. These baseline results are presented in Table 3 and will be later assessed in this paper, in the Results and discussion section.

**Table 3:** Baseline results for cancer detection, using a vanilla AE with random weights

	Top Layers (AE)	Accuracy (%)	MCC	Precision (%)	Recall (%)	Fscore (%)
Thyroid	AE: Encoding Layers	83.03 ±2.17	0.16 ±0.16	52.46 ±38.70	15.10 ±18.39	18.80 ±19.71
	AE: Complete AE	93.07 ±1.52	0.76 ±0.04	81.12 ±9.41	79.58 ±8.46	79.57 ±3.87
Skin	AE: Encoding Layers	82.87 ±2.77	0.23 ±0.10	43.46 ±10.55	25.00 ±9.75	30.98 ±9.73
	AE: Complete AE	87.47 ±4.28	0.54 ±0.06	64.80 ±12.79	59.79 ±9.38	60.55 ±5.11
Stomach	AE: Encoding Layers	84.63 ±2.41	0.19 ±0.06	42.11 ±9.80	17.33 ±7.71	22.90 ±7.37
	AE: Complete AE	87.40 ±2.68	0.47 ±0.10	55.77 ±10.29	51.66 ±8.72	53.24 ±8.33
Breast	AE: Encoding Layers	82.13 ±4.16	0.22 ±0.10	53.51 ±20.92	20.60 ±12.96	25.94 ±13.06
	AE: Complete AE	87.00 ±1.58	0.52 ±0.04	62.81 ±7.03	57.80 ±6.29	59.70 ±3.35
Lung	AE: Encoding Layers	81.60 ±1.26	0.15 ±0.07	40.78 ±11.11	14.88 ±8.74	20.45 ±9.95
	AE: Complete AE	85.30 ±3.50	0.50 ±0.06	59.78 ±11.76	59.11 ±9.72	57.99 ±4.88

All the presented results are the 10-fold cross-validation mean values, at the validation set, by selecting the best performing model according to its *F*_1_ *score*

**Table 4:** Performance comparison when using each of the 3 AEs — Basic AE, Denoising AE and Sparse AE — and for each type of cancer

	Top Layers (AEs)	Accuracy (%)	MCC	Precision (%)	Recall (%)	F_1_ score
**Fixing the AE weights (Approach A)**						
Thyroid	AE: Encoding Layers	91.80 ±3.34	0.72 ±0.11	76.06 ±11.72	78.98 ±12.01	76.64 ±8.98
	AE: Complete Autoencoder	94.90 ±1.56	0.82 ±0.06	84.13 ±5.00	86.43 ±6.63	85.32 ±4.57
	DAE: Encoding Layers	87.63 ±3.49	0.57 ±0.09	67.41 ±12.33	63.48 ±18.10	63.49 ±9.42
	DAE: Complete Autoencoder	94.03 ±1.06	0.79 ±0.04	81.79 ±4.60	84.37 ±4.87	83.11 ±2.73
	SAE: Encoding Layers	87.93 ±2.66	0.57 ±0.14	65.43 ±7.70	64.25 ±22.35	62.41 ±14.32
	SAE: Complete Autoencoder	92.67 ±1.96	0.75 ±0.06	77.13 ±8.76	82.51 ±4.57	79.42 ±4.72
Skin	AE: Encoding Layers	90.77 ±3.52	0.63 ±16.17	74.71 ±13.54	61.40 ±17.15	66.83 ±15.08
	AE: Complete Autoencoder	92.40 ±2.65	0.69 ±0.12	80.92 ±9.21	67.35 ±13.36	73.12 ±11.00
	DAE: Encoding Layers	85.07 ±4.16	0.42 ±0.16	56.22 ±16.42	47.02 ±18.01	48.76 ±14.72
	DAE: Complete Autoencoder	89.43 ±1.32	0.58 ±0.06	69.42 ±5.65	60.15 ±8.18	63.98 ±5.04
	SAE: Encoding Layers	79.27 ±3.70	0.07 ±5.71	33.49 ±25.49	13.79 ±7.76	16.11 ±8.02
	SAE: Complete Autoencoder	85.83 ±2.04	0.45 ±0.07	56.34 ±7.81	49.57 ±6.24	52.42 ±5.64
Stomach	AE: Encoding Layers	91.60 ±1.91	0.62 ±0.09	76.09 ±9.45	58.07 ±10.87	65.35 ±8.96
	AE: Complete Autoencoder	94.00 ±1.47	0.74 ±0.07	83.45 ±8.34	71.56 ±6.24	76.78 ±5.42
	DAE: Encoding Layers	86.50 ±4.33	0.33 ±0.16	64.35 ±22.75	27.46 ±12.98	34.93 ±15.68
	DAE: Complete Autoencoder	91.03 ±1.57	0.58 ±0.08	74.03 ±7.85	54.91 ±9.42	62.61 ±7.69
	SAE: Encoding Layers	85.93 ±1.34	0.16 ±0.11	48.24 ±25.25	11.06 ±6.73	17.49 ±9.35
	SAE: Complete Autoencoder	89.87 ±2.06	0.56 ±8.23	66.10 ±9.38	57.09 ±6.33	61.02 ±6.82
Breast	AE: Encoding Layers	88.40 ±5.52	0.59 ±0.17	68.39 ±19.13	64.80 ±10.84	65.91 ±13.72
	AE: Complete Autoencoder	91.77 ±3.13	0.69 ±0.12	80.57 ±11.79	67.00 ±11.24	72.91 ±10.86
	DAE: Encoding Layers	83.53 ±1.74	0.25 ±0.14	51.39 ±25.04	25.60 ±15.57	31.23 ±17.51
	DAE: Complete Autoencoder	87.30 ±1.90	0.53 ±0.05	63.43 ±7.13	58.60 ±5.17	60.67 ±4.58
	SAE: Encoding Layers	79.73 ±3.86	0.02 ±0.05	9.80 ±12.48	3.00 ±3.16	4.11 ±4.09
	SAE: Complete Autoencoder	84.07 ±2.40	0.41 ±0.07	53.13 ±8.05	47.80 ±4.85	50.13 ±5.62

For the malaria dataset, we consider two results of two different approaches, applied to the same domain. Firstly, in [31], the authors used a support vector machine (SVM) to automatically classify each species-stage combination of the malaria parasite. The authors studied the SVM hyperparameters and their influence on the classifier’s performance. When considering F_1_ score, this classifier performance ranged from 18.8% to 87.4%, considering all the malaria parasite species-stage combinations. Secondly, in [46] a 5-class MobileNet v2 convolutional neural network was used to directly classify the thin blood smears images. The chosen architecture presented an F_1_ score of 53% when detecting parasites from artifacts.

For the Wisconsin Breast Cancer dataset, we chose as baseline the work presented in [47], where the authors studied different machine learning algorithms, combined with a Principal Component Analysis (PCA) to detect tumorous and non-tumorous samples on this dataset. Furthermore, they compared their best top 3 models with some state-of-the-art models. Their overall best was the combination a Naïve Bayes with a Sigmoid PCA, with an F_1_ score of approximately 97%.

## Results and discussion

**Autoencoders as weight initialization can efficiently predict diseases when applied to different biological and feature-extracted data.** Given the results, one tends to assume that the methodology originally presented in [19] generalizes to different data and problems. This work can be seen as another empirical proof supporting this premise. We outperform the results of Ferreira et al. [21] and the baseline results presented in Tables 2 and 3; our best performance was achieved by combining the pre-trained AE encoding layers import to the upper layers (Strategy 1) of the deep classification network and allowing subsequent *fine-tuning* (Approach B), with an F_1_ score of 99.03 and an MCC of 0.99, when distinguishing thyroid from the other cancer types (and an average F_1_ score of 98.27%, when considering all cancer classifications). The various networks combinations also achieved very high results for each cancer type, as observable in Table 4. Furthermore, our methodology outperformed the established baselines for both image-based features datasets. The best overall performances were:

- The combination of the pre-trained DAE encoding layers import to the upper layers (Strategy 1) of the deep classification network and allowing subsequent *fine-tuning* (Approach B), with an F_1_ score of 89.95% and an MCC of 0.84, on the Malaria dataset (as highlighted in Table 5);

**Table 4:** Performance comparison when using each of the 3 AEs — Basic AE, Denoising AE and Sparse AE — and for each type of cancer *(Continued)*

Lung	AE: Encoding Layers	85.97 ±7.00	0.54 ±0.13	65.00 ±17.54	61.25 ±12.30	60.94 ±11.01
	AE: Complete Autoencoder	90.93 ±2.56	0.67 ±0.09	77.28 ±9.43	66.90 ±8.26	71.51 ±7.94
	DAE: Encoding Layers	81.77 ±3.17	0.25 ±0.13	45.70 ±25.30	28.38 ±16.21	32.15 ±15.21
	DAE: Complete Autoencoder	85.73 ±3.28	0.49 ±0.09	60.30 ±9.76	53.40 ±7.49	56.21 ±7.44
	SAE: Encoding Layers	79.70 ±3.66	0.11 ±0.08	23.94 ±30.04	4.88 ±3.81	7.13 ±5.27
	SAE: Complete Autoencoder	83.23 ±2.59	0.40 ±0.09	51.33 ±7.62	49.33 ±10.08	49.83 ±7.52
**Fine-Tuning the AE Weights (Approach B)**						
Thyroid	AE: Encoding Layers	**99.67 ±0.42**	**0.99 ±0.01**	**98.29 ±2.09**	**99.80 ±0.62**	**99.03 ±1.21**
	AE: Complete Autoencoder	99.67 ±0.22	0.99 ±0.01	99.22 ±1.00	98.82 ±1.02	99.02 ±0.65
	DAE: Encoding Layers	99.57 ±0.55	0.99 ±0.02	97.77 ±3.08	99.80 ±0.62	98.75 ±1.56
	DAE: Complete Autoencoder	99.60 ±0.38	0.99 ±0.01	99.22 ±1.01	98.42 ±2.05	98.81 ±1.15
	SAE: Encoding Layers	95.47 ±1.01	0.85 ±0.02	80.98 ±4.76	96.47 ±3.31	87.90 ±2.20
	SAE: Complete Autoencoder	97.73 ±0.52	0.93 ±0.02	89.39 ±2.69	98.43 ±2.03	93.65 ±1.41
Skin	AE: Encoding Layers	99.50 ±0.32	0.98 ±0.01	98.12 ±1.52	98.73 ±1.48	98.45 ±1.01
	AE: Complete Autoencoder	99.33 ±0.57	0.97 ±0.02	99.35 ±1.45	96.41 ±2.99	97.84 ±1.84
	DAE: Encoding Layers	99.30 ±0.51	0.97 ±0.02	97.52 ±2.12	98.09 ±2.34	97.78 ±1.62
	DAE: Complete Autoencoder	99.50 ±0.53	0.98 ±0.02	99.58 ±0.89	97.24 ±3.48	98.36 ±1.77
	SAE: Encoding Layers	95.80 ±1.18	0.84 ±0.05	93.23 ±5.06	79.43 ±7.22	85.51 ±4.38
	SAE: Complete Autoencoder	97.53 ±1.08	0.90 ±0.05	95.76 ±2.83	88.37 ±7.12	91.74 ±3.94
Stomach	AE: Encoding Layers	99.43 ±0.39	0.98 ±1.71	98.21 ±1.70	97.83 ±1.36	97.98 ±1.36
	AE: Complete Autoencoder	99.17 ±0.59	0.97 ±0.02	97.60 ±1.98	96.39 ±4.24	96.93 ±2.26
	DAE: Encoding Layers	99.33 ±0.47	0.97 ±0.02	97.84 ±2.10	97.35 ±2.39	97.57 ±1.72
	DAE: Complete Autoencoder	99.23 ±0.57	0.97 ±0.02	98.08 ±1.90	96.35 ±3.86	97.16 ±2.16
	SAE: Encoding Layers	95.60 ±0.81	0.81 ±0.04	93.33 ±3.92	73.72 ±7.08	82.12 ±3.96
	SAE: Complete Autoencoder	97.37 ±0.55	0.89 ±2.89	96.08 ±3.01	84.56 ±4.90	89.83 ±2.43
Breast	AE: Encoding Layers	99.33 ±0.52	0.98 ±0.02	97.85 ±2.32	98.20 ±1.48	98.01 ±1.55
	AE: Complete Autoencoder	99.30 ±0.37	0.98 ±0.01	99.00 ±1.06	96.80 ±2.35	97.87 ±1.15
	DAE: Encoding Layers	99.20 ±0.65	0.97 ±0.02	97.83 ±2.54	97.40 ±1.90	97.60 ±1.95
	DAE: Complete Autoencoder	99.23 ±0.52	0.97 ±0.02	98.60 ±2.08	96.80 ±1.69	97.68 ±1.57
	SAE: Encoding Layers	96.70 ±1.24	0.89 ±0.05	95.29 ±4.78	84.60 ±6.47	89.45 ±4.14
	SAE: Complete Autoencoder	97.40 ±1.12	0.90 ±0.04	95.78 ±4.02	88.40 ±4.79	91.87 ±3.52
Lung	AE: Encoding Layers	99.27 ±0.83	0.97 ±0.03	97.34 ±3.08	98.44 ±2.02	97.87 ±2.40
	AE: Complete Autoencoder	99.23 ±0.45	0.97 ±0.02	98.83 ±1.63	96.67 ±2.46	97.71 ±1.34
	DAE: Encoding Layers	99.00 ±0.75	0.96 ±0.03	96.89 ±2.27	97.26 ±2.65	97.06 ±2.23
	DAE: Complete Autoencoder	99.27 ±0.52	0.97 ±0.02	97.95 ±2.69	97.85 ±3.12	97.87 ±1.58
	SAE: Encoding Layers	95.27 ±1.43	0.82 ±0.06	90.69 ±4.64	80.61 ±6.72	85.21 ±4.78
	SAE: Complete Autoencoder	97.00 ±0.96	0.89 ±0.04	93.65 ±2.56	88.44 ±5.36	90.88 ±3.19

When measuring loss, lower is better. For all the remaining metrics, higher is better. All the presented results are the 10-fold cross-validation mean values, at the validation set, by selecting the best performing model according to its *F*_1_ *score*. The highlighted values correspond to the combination that led to the overall best result (detecting thyroid cancer, importing only the encoding layers a Basic AE into the classification network, and allowing subsequent fine-tune, when training for the classification task)

**Table 5:** Performance comparison when using each of the 3 AEs — Basic AE, Denoising AE and Sparse AE — and for malaria detection

	Top Layers (AE)	Accuracy (%)	MCC	Precision (%)	Recall (%)	F_1_ score
Approach A	AE: Encoding Layers	62.82 ±0.60	0.03 ±0.04	66.17 ±41.60	1.90 ±3.97	3.32 ±6.49
	AE: Complete Autoencoder	62.73 ±0.45	0.03 ±0.04	53.97 ±34.95	2.12 ±4.23	3.66 ±6.72
	DAE: Encoding Layers	62.50 ±0.07	0.00 ±0.00	0.00 ±0.00	0.00 ±0.00	0.00 ±0.00
	DAE: Complete Autoencoder	62.50 ±0.07	0.00 ±0.00	0.00 ±0.00	0.00 ±0.00	0.00 ±0.00
	SAE: Encoding Layers	62.21 ±0.34	-0.01 ±0.02	13.17 ±17.36	0.23 ±0.32	0.44 ±0.63
	SAE: Complete Autoencoder	62.51 ±0.07	0.21 ±0.65	10.00 ±31.62	0.05 ±0.14	0.09 ±0.28
Approach B	AE: Encoding Layers	91.28 ±1.17	0.82 ±0.02	87.41 ±2.69	89.84 ±3.32	88.53 ±1.58
	AE: Complete Autoencoder	91.43 ±1.21	0.82 ±0.02	88.12 ±2.37	89.25 ±2.21	88.66 ±1.58
	DAE: Encoding Layers	**92.36 ±0.46**	**0.84 ±0.01**	**88.91 ±2.14**	**91.11 ±2.49**	**89.95 ±0.61**
	DAE: Complete Autoencoder	92.18 ±0.83	0.84 ±0.02	88.54 ±2.33	91.19 ±1.68	89.78 ±1.00
	SAE: Encoding Layers	62.21 ±0.34	-0.01 ±0.02	13.17 ±17.36	0.23 ±0.32	0.44 ±0.63
	SAE: Complete Autoencoder	62.51 ±0.07	0.01 ±0.01	10.00 ±31.62	0.05 ±0.14	0.09 ±0.28

All the presented results are the 10-fold cross-validation mean values, at the validation set, by selecting the best performing model according to its *F*_1_ *score*. The first row presents the results for Approach A, where we fix the resulting weights of the AE pre-training; the second one shows the results for Approach B, where we allow the subsequent fine-tuning of all the weights of the model. The highlighted values correspond to the combination that led to the overall best result (importing only the encoding layers a Denoising AE into the classification network, and allowing subsequent fine-tune, when training for the classification task)
- The combination of the pre-trained AE encoding layers import to the upper layers (Strategy 1) of the deep classification network and allowing subsequent *fine-tuning* (Approach B), with an F_1_ score of 98.84% and an MCC of 0.98, on the Wisconsin Breast Cancer dataset, (as shown in Table 6).

**Table 6:** Performance comparison when using each of the 3 AEs — Basic AE, Denoising AE and Sparse AE — and for breast cancer detection, on the UCI’s Wisconsin Breast Cancer dataset

	Top Layers (AEs)	Accuracy (%)	MCC	Precision (%)	Recall (%)	F_1_ score
Approach A	AE: Encoding Layers	97.54 ±2.06	0.95 ±0.04	98.67 ±2.92	94.81 ±5.20	96.60 ±2.90
	AE: Complete Autoencoder	96.49 ±2.62	0.93 ±0.05	96.83 ±3.71	93.83 ±7.12	95.11 ±3.93
	DAE: Encoding Layers	95.43 ±3.81	0.90 ±0.08	98.38 ±3.48	89.13 ±8.73	93.36 ±6.05
	DAE: Complete Autoencoder	93.32 ±3.78	0.86 ±0.08	98.19 ±2.93	83.46 ±8.52	90.09 ±5.98
	SAE: Encoding Layers	97.19 ±2.22	0.94 ±0.05	97.69 ±3.15	94.81 ±5.20	96.14 ±3.13
	SAE: Complete Autoencoder	97.02 ±2.35	0.94 ±0.05	97.70 ±2.42	94.31 ±7.03	95.80 ±3.64
Approach B	AE: Encoding Layers	**99.12 ±1.24**	**0.98 ±0.03**	**98.71 ±2.86**	**99.05 ±2.01**	**98.84 ±1.59**
	AE: Complete Autoencoder	98.60 ±1.38	0.97 ±0.03	97.75 ±2.38	98.57 ±3.21	98.11 ±1.91
	DAE: Encoding Layers	97.72 ±2.62	0.95 ±0.06	98.08 ±2.50	95.74 ±6.13	96.81 ±3.83
	DAE: Complete Autoencoder	97.19 ±2.64	0.94 ±0.06	96.39 ±4.57	96.23 ±4.91	96.22 ±3.62
	SAE: Encoding Layers	97.19 ±2.22	0.94 ±0.04	96.15 ±5.24	96.71 ±3.19	96.31 ±2.78
	SAE: Complete Autoencoder	96.66 ±2.10	0.93 ±0.04	97.66 ±3.28	93.44 ±5.47	95.39 ±2.96

All the presented results are the 10-fold cross-validation mean values, at the validation set, by selecting the best performing model according to its *F*_1_ *score*. The first row presents the results for Approach A, where we fix the resulting weights of the AE pre-training; the second one shows the results for Approach B, where we allow the subsequent fine-tuning of all the weights of the model. The highlighted values correspond to the combination that led to the overall best result (importing only the encoding layers a Basic AE into the classification network, and allowing subsequent fine-tune, when training for the classification task.)

With these results, there is evidence that this methodology can generalize to other types of data and tasks.

**Subsequent model fine-tuning (Approach B) leads to better results than fixing the weights (Approach A).** Similarly to [19], it was clear that, with the new data, our results for all the experiments in the three datasets support that allowing the imported weights of the AEs to be fine-tuned in the training phase gave better results than fixing them.

**There is high evidence supporting that importing only the encoding part of the AE leads to good results.** According to the results in Table 7, and considering Approach A, the Strategy 1 of embedding with extra random decoding yielded better results in comparison to Strategy 2, for all the combination except when using an SAE. Regarding Approach B, all combinations achieved quite close results for all the performed experiments. Thus, one can argue that less complex models can achieve better results, similar to what was concluded in [21].

**Table 7:** Performance comparison when adding a decoder layer with random weights when using Strategy 1 (importing only the enconder part of AE), for each of the 3 AEs — Basic AE, Denoising AE and Sparse AE — for breast cancer detection, with RNA-Seq input

	Top Layers (AEs)	Accuracy (%)	MCC	Precision (%)	Recall (%)	F_1_ score
Approach A	AE: Encoding Layer (n =2)	88.40 ±5.52	0.59 ±0.17	68.39 ±19.13	64.80 ±10.84	65.91 ±13.72
	AE: Complete Autoencoder	91.77 ±3.13	0.69 ±0.12	80.57 ±11.79	67.00 ±11.24	72.91 ±10.86
	AE: Encoding Layer (n =3)	92.53 ±2.25	0.72 ±0.09	80.75 ±7.45	72.31 ±11.29	76.50 ±8.12
	DAE: Encoding Layer (n =2)	83.53 ±1.74	0.25 ±0.14	51.39 ±25.04	25.60 ±15.57	31.23 ±17.51
	DAE: Complete Autoencoder	87.30 ±1.90	0.53 ±0.05	63.43 ±7.13	58.60 ±5.17	60.67 ±4.58
	DAE: Encoding Layer (n =3)	87.47 ±2.81	0.57 ±0.08	62.88 ±10.52	68.00 ±8.99	64.51 ±6.24
	SAE: Encoding Layer (n =2)	79.73 ±3.86	0.02 ±0.05	9.80 ±12.48	3.00 ±3.16	4.11 ±4.09
	SAE: Complete Autoencoder	84.07 ±2.40	0.41 ±0.07	53.13 ±8.05	47.80 ±4.85	50.13 ±5.62
	SAE: Encoding Layer (n =3)	76.33 ±8.91	0.36 ±0.11	41.26 ±12.14	62.20 ±12.80	47.83 ±8.30
Approach B	AE: Encoding Layer (n =2)	99.33 ±0.52	0.98 ±0.02	97.85 ±2.32	98.20 ±1.48	98.01 ±1.55
	AE: Complete Autoencoder	99.30 ±0.37	0.98 ±0.01	99.00 ±1.06	96.80 ±2.35	97.87 ±1.15
	AE: Encoding Layer (n =3)	99.17 ±0.53	0.97 ±0.02	98.43 ±1.98	96.60 ±3.27	97.46 ±1.65
	DAE: Encoding Layer (n =2)	99.20 ±0.65	0.97 ±0.02	97.83 ±2.54	97.40 ±1.90	97.60 ±1.95
	DAE: Complete Autoencoder	99.23 ±0.52	0.97 ±0.02	98.60 ±2.08	96.80 ±1.69	97.68 ±1.57
	DAE: Encoding Layer (n =3)	99.33 ±0.38	0.98 ±0.01	99.20 ±1.40	96.80 ±1.69	98.02 ±1.08
	SAE: Encoding Layer (n =2)	96.70 ±1.24	0.89 ±0.05	95.29 ±4.78	84.60 ±6.47	89.45 ±4.14
	SAE: Complete Autoencoder	97.40 ±1.12	0.90 ±0.04	95.78 ±4.02	88.40 ±4.79	91.87 ±3.52
	SAE: Encoding Layer (n =3)	97.27 ±0.64	0.90 ±0.02	93.58 ±1.91	89.80 ±3.71	91.61 ±2.09

The first row presents the results for Approach A, where we fix the resulting weights of the AE pre-training; the second one shows the results for Approach B, where we allow the subsequent fine-tuning of all the weights of the model. All the presented results are the 10-fold cross-validation mean values, at the validation set, by selecting the best performing model according to its *F*_1_ *score*. *n* represents the number of layers of the encoder

**There is no evidence of overfitting, considering the additional experiments with held-out test sets.** According to the results in Table 8, which are representative of the models’ evaluation on two distinct held-out test sets, one can affirm that our models generalize well to new data. The results in the test phase were similar to the ones on the validation sets in the training phase (a difference of approximately 3% and 2% respectively, for both the Malaria and the Wisconsin Breast Cancer data), meaning also that the models do not seem to overfit the training data.

**Table 8:** Performance comparison when using the vanilla AE on two held-out test sets (Malaria and Wisconsin Breast Cancer, respectively)

	Top Layers (AEs)	Accuracy (%)	MCC	Precision (%)	Recall (%)	F_1_ score
Train	M: Encoding Layers	91.21% ±1.56%	0.81 ±0.03	86.61% ±3.77%	90.84% ±2.91%	88.59% ±1.86%
	M: Complete Autoencoder	90.19% ±2.08%	0.80 ±0.04	85.29% ±4.69%	89.69% ±2.83%	87.33% ±2.33%
	WBC: Encoding Layers	98.69% ±1.38%	0.97 ±0.03	99.37% ±1.98%	97.14% ±3.69%	98.20% ±1.91%
	WBC: Complete Autoencoder	97.90% ±2.07%	0.96 ±0.04	95.54% ±5.18%	99.29% ±2.26%	97.28% ±2.65%
Test	M: Encoding Layers	89.64%	0.78	90.02%	81.40%	85.49%
	M: Complete Autoencoder	86.10%	0.70	82.86%	79.34%	81.06%
	WBC: Encoding Layers	97.34%	0.95	99.99%	92.86%	96.30%
	WBC: Complete Autoencoder	95.74%	0.91	98.44%	90.00%	94.03%

The presented results in the first row (Train) are the 10-fold cross-validation mean values, at the validation set, by selecting the best performing model according to its *F*_1_ *score*. The second row (Test) gathers the results when evaluating the models on the testing phase. For both datasets, two thirds of the data were used in the training phase, and one third as the held-out in the test phase. *M* represents the Malaria dataset, and *WBC* the Wisconsin Breast Cancer one

**Changing the AE latent vector dimension has more impact when fixing the AE pre-trained weights (Approach A) than when fine-tuning all the weights (Approach B).** When varying the latent vector dimension, the 3 tested dimensions achieved lower results than the encoding layer size used as default when analyzing Approach A (Fixed Weights). However, when analyzing Approach B (Fine-Tuning Weights), the results in Table 9 show no significant variation in the DNN performance, for both the embedding AE strategies, with a F_1_ score variation of 1% to 3%, comparing with the default size experiment. In Approach A (Fixing Weights), the performance difference was more significant, with the F_1_ score decreasing nearly 20% with a latent vector dimension of 64, and approximately 60% with a dimension of 16, for Strategy 1.

**Table 9:** Performance comparison of the classifier, for the Basic AE, when varying the dimension of its latent vector, in the RNA-Seq input

Dim	Top Layers (AEs)	Accuracy (%)	MCC	Precision (%)	Recall (%)	F_1_ score
**Fixing the AE weights (Approach A)**						
128 ^∗^	AE: Encoding Layers	88.40 ±5.52	0.59 ±0.17	68.39 ±19.13	64.80 ±10.84	65.91 ±13.72
	AE: Complete AE	91.77 ±3.13	0.69 ±0.12	80.57 ±11.79	67.00 ±11.24	72.91 ±10.86
64	AE: Encoding Layers	84.83 ±3.05	0.37 ±0.13	59.08 ±15.04	36.40 ±11.23	44.12 ±10.52
	AE: Complete AE	88.37 ±3.61	0.56 ±0.14	67.58 ±13.26	59.20 ±10.96	62.94 ±11.39
32	AE: Encoding Layers	84.10 ±2.12	0.22 ±0.16	54.76 ±20.42	15.60 ±14.20	22.55 ±17.08
	AE: Complete AE	86.13 ±2.34	0.48 ±0.09	59.22 ±6.90	54.00 ±10.20	56.23 ±8.17
16	AE: Encoding Layers	83.87 ±0.67	0.09 ±0.10	43.75 ±47.60	4.40 ±5.95	7.66 ±9.82
	AE: Complete AE	84.17 ±3.23	0.42 ±0.12	52.95 ±11.05	50.00 ±11.89	51.04 ±10.61
**Fine-Tuning the AE Weights (Approach B)**						
128 ^∗^	AE: Encoding Layers	99.33 ±0.52	0.98 ±0.02	97.85 ±2.32	98.20 ±1.48	98.01 ±1.55
	AE: Complete AE	99.30 ±0.37	0.98 ±0.01	99.00 ±1.06	96.80 ±2.35	97.87 ±1.15
64	AE: Encoding Layers	99.43 ±0.50	0.98 ±0.02	97.86 ±2.12	98.80 ±1.69	98.31 ±1.49
	AE: Complete AE	99.30 ±0.29	0.97 ±0.01	98.62 ±1.61	97.20 ±2.15	97.88 ±0.90
32	AE: Encoding Layers	99.03 ±0.55	0.97 ±0.02	97.23 ±2.12	97.00 ±2.54	97.09 ±1.67
	AE: Complete AE	99.07 ±0.54	0.97 ±0.02	98.59 ±1.35	95.80 ±3.46	97.13 ±1.71
16	AE: Encoding Layers	98.80 ±0.74	0.96 ±0.02	96.51 ±3.53	96.40 ±1.84	96.42 ±2.16
	AE: Complete AE	98.70 ±0.43	0.95 ±0.01	97.78 ±1.99	94.40 ±2.63	96.02 ±1.33

The experiment pipeline remains the same, under the same evaluation metrics. The *Dim* column represents the latent vector dimension. The ∗ symbol represents the dimension used as default

**There is no evidence supporting a conclusion on which is the best data imputation strategy.** After the imputation strategy experiment, the results pointed out that the mean strategy led to the highest performance in the classification task when considering Approach B. However, one can observe in Table 10 that the mode strategy yielded better results for Approach A, but all the other imputation strategies achieved similar results. Hence, we cannot affirm that there is a particular strategy that leads to better classification results. Further studies should be considered on the RNA-Seq data preprocessing step to support this claim.

**Table 10:** Performance comparison of the classifier, for the Basic AE, when changing the imputation strategy at the data preprocessing step

Strategy	Top Layers (AEs)	Accuracy (%)	MCC	Precision (%)	Recall (%)	F_1_ score
**Fixing the AE weights (Approach A)**						
Mean ^∗^	AE: Encoding Layers	88.40 ±5.52	0.59 ±0.17	68.39 ±19.13	64.80 ±10.84	65.91 ±13.72
	AE: Complete AE	91.77 ±3.13	0.69 ±0.12	80.57 ±11.79	67.00 ±11.24	72.91 ±10.86
CV	AE: Encoding Layers	91.93 ±2.13	0.69 ±0.10	79.43 ±6.20	69.40 ±10.96	73.81 ±8.14
	AE: Complete AE	93.23 ±1.99	0.74 ±0.08	83.41 ±5.85	74.20 ±9.59	78.31 ±6.95
MFV	AE: Encoding Layers	92.50 ±2.36	0.71 ±0.10	82.60 ±7.41	70.00 ±13.40	75.16 ±9.23
	AE: Complete AE	93.27 ±1.71	0.74 ±0.07	84.97 ±4.01	72.40 ±9.74	77.91 ±6.54
**Fine-Tuning the AE Weights (Approach B)**						
Mean ^∗^	AE: Encoding Layers	99.33 ±0.52	0.98 ±0.02	97.85 ±2.32	98.20 ±1.48	98.01 ±1.55
	AE: Complete AE	99.30 ±0.37	0.98 ±0.01	99.00 ±1.06	96.80 ±2.35	97.87 ±1.15
CV	AE: Encoding Layers	99.40 ±0.49	0.98 ±0.02	98.63 ±2.04	97.80 ±2.39	98.23 ±1.48
	AE: Complete AE	99.30 ±0.53	0.98 ±0.02	99.01 ±1.39	96.80 ±3.29	97.97 ±1.38
MFV	AE: Encoding Layers	99.47 ±0.32	0.98 ±0.01	98.83 ±1.64	98.00 ±2.11	98.39 ±0.98
	AE: Complete AE	99.13 ±0.57	0.97 ±0.02	98.77 ±1.71	96.00 ±2.31	97.36 ±1.74

The experiment pipeline remains the same, under the same evaluation metrics. The *Strategy* column represents the imputation strategy used. The ∗ symbol represents the default strategy. The following abreviations were used: *CV* for Constante Value, and *MFV* for Most Frequent Value

**Fig. 2:**
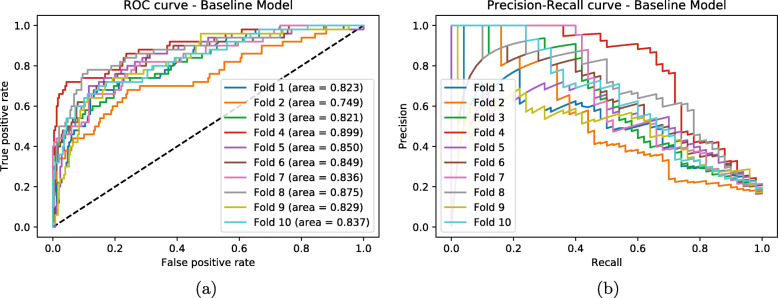
Baseline ROC-AUC and Precision-Recall Curves for the RNA-Seq data. Baseline **a** ROC-AUC and **b** Precision-Recall curves when detecting breast cancer detection in the RNA-Seq input data, using a Fully Connected Neural Network (the classification architecture, without the AE as top layers). Each line represent the different 10 folds for the cross validation

**Fig. 3:**
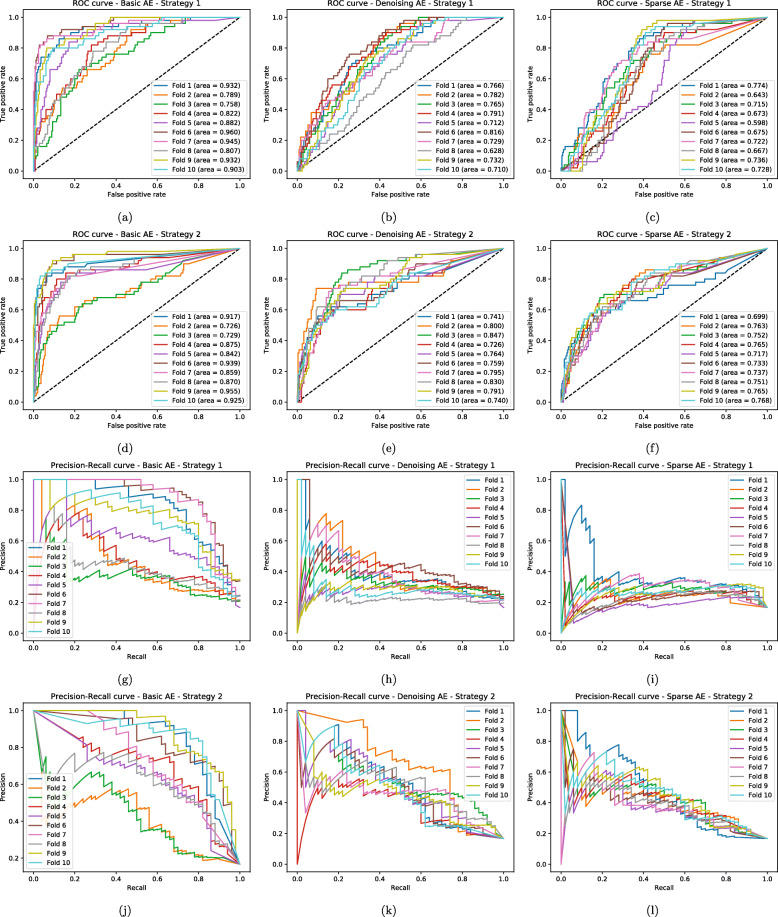
ROC-AUC and Precision-Recall Curves for the RNA-Seq data, with Approach A. Classification Model ROC-AUC — **a** to **f** — and **b** Precision-Recall — **g** to **l** — curves when detecting breast cancer detection in the RNA-Seq input data, with Approach A (fixing the weights imported from the AE) combined with both AE importing strategies. Each line represent the different 10 folds for the cross validation

**Fig. 4:**
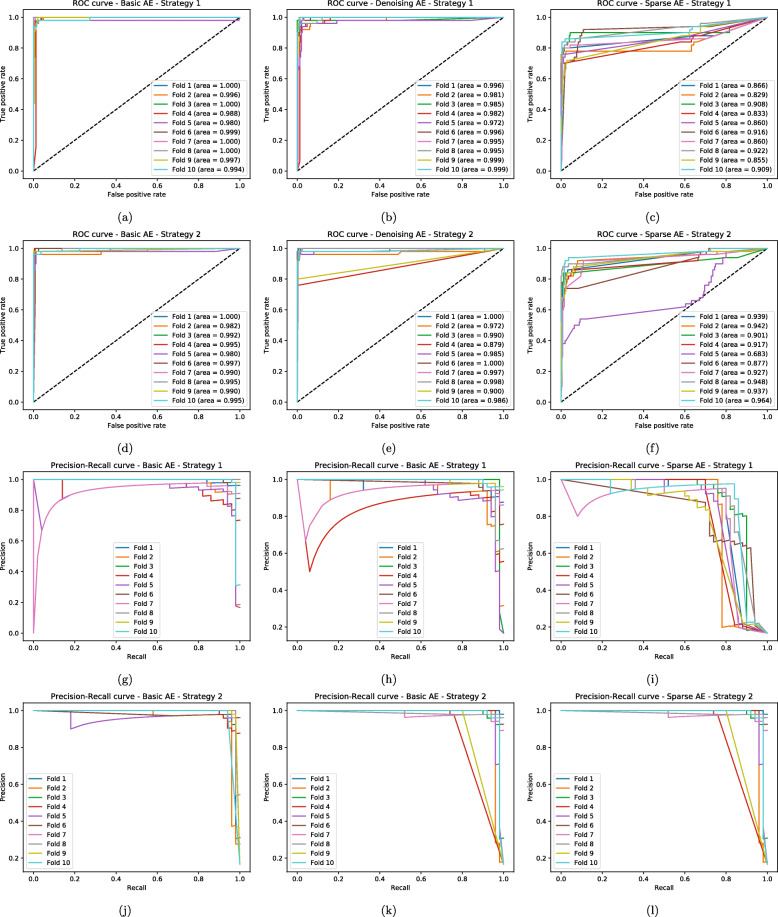
ROC-AUC and Precision-Recall Curves for the RNA-Seq data, with Approach B. Classification Model ROC-AUC — **a** to **f** — and **b** Precision-Recall — **g** to **l** — curves when detecting breast cancer detection in the RNA-Seq input data, with Approach B (allowing fine tune on the weights imported from the AE) combined with both AE importing strategies. Each line represent the different 10 folds for the cross validation

It is possible to observe the complete results for all the experiments in Tables 2, 3, 4, 5, 6, 7, 8, 9 and 10, and also in Figs. 2, 3 and 4. Since detecting thyroid cancer with the Basic AE’s encoding part initialization was the combination with the best overall results, for the experiments summarized in Tables 7, 8, 9 and 10 and Figs. 2, 3 and 4 we used that specific AE to assess if there were changes in the classification network performance. However, due to space constraints, we opted to only present the results for the breast cancer class, since it had a greater results variance between strategies, especially for Approach A, as seen in Table 4.

## Conclusions

We compared the performance of a deep neural network (DNN) when using three different autoencoders (AEs) to initialize its weights. To do so, each AE was pre-trained and then attached to the top layers of our classifier. In the importation phase, two different strategies were studied: (1) just importing the AE’s encoding layer, and (2) importing all the AE’s layers. Each of the three built architectures was then trained to classify the input data as one of the five types of cancer in this study. Two different approaches were analyzed, in the training process: (A) fixing the imported weights, and (B) by allowing them to be fine-tuned during supervised training. Additionally, we studied (1) how changing the encoding space dimension impacts the AEs and DNN performances, and (2) how the missing data replacement strategy influences the performance in the classification task. We also assessed the impact that the number of AE imported layers has on the DNN overall performance.

Furthermore, we extended the generalization study of this methodology by applying it to two different datasets: the MalariaScope thin blood smears data and the Wisconsin Breast Cancer tumors datasets.

We outperformed the best result reported in [21], according not just to the F_1_ score, but to all the other evaluation metrics as well. After a 10-fold cross-validation training process, a full embedding of a pre-trained Basic AE to the top layers of the DNN (Strategy 2), followed by fine-tuning, achieved the best overall performance, with an F_1_ score of 99.03 ±1.21. Moreover, we outperformed as well other established baselines, for the MalariaScope and Wisconsin Breast Cancer datasets, supporting the claim that this methodology generalizes well, including when dealing with other data types. After performing two distinct held-out datasets, we could conclude that our models generalize well to unseen and different data, not overfitting during the training phase. Allowing fine-tune (Approach B) on the imported weights of the AEs led undeniably to better results than fixing the weights of the top layers (Approach A), as can be observed in the results. Approach A is more sensitive to latent vector dimension variations, in comparison with a more stable Approach B. Finally, the results showed no evidence on which imputation strategy is the best, considering the RNA-Seq data.

In conclusion, this methodology led to state-of-the-art performance in cancer classification from gene expression, strongly supporting that using AE as weight initialization can help DNNs achieving better performances. We believe that it also has high potential of generalizing well to other data and problems, as shown in the results using datasets of features extracted from images.

In the long term, and although some of the data is considered a toy dataset, we expect that this work will lead to a more efficient and robust automated system for the diagnosis of diseases, in particular cancer, providing a faster diagnostic, and improving the expected treatment outcome.

## Data Availability

The datasets analysed during the current study are available in the cBioPortal repository (https://www.cbioportal.org/datasets), and in the Machine Learning Repository of the University of California Irvine (https://archive.ics.uci.edu/ml/datasets/Breast+Cancer+Wisconsin+(Diagnostic)). All the RNA-Seq datasets are from the *TCGA PanCancer Atlas* project. The Malaria dataset is not public; it belongs to the Fraunhofer Portugal’s MalariaScope project.

## Abbreviations

AEs: Autoencoders
AUC: Area under the curve
DAE: Denoising autoencoder
DNN: Deep neural network
GDL: Genome deep learning
ICGC: The international cancer genome consortium
KPCA: Kernel principal component analysis
MCC: Matthews correlation coefficient
MLP-SAE: Multilayer perceptron and stacked denoising autoencoder
MSE: Mean squared error
PCA: Principal component analysis
ReLU: Rectified linear units
ROC: Receive operating characteristic
SAE: Sparse autoencoder
SSAE: Stacked sparse autoencoder
SVM: Support vector machine
TCGA: The cancer genome atlas
